# Robotic Radiosurgery for Sacral Chordoma: Preserving Function in a Clinical Conundrum Scenario

**DOI:** 10.7759/cureus.83747

**Published:** 2025-05-08

**Authors:** Alejandro Gonzalez-Motta, Diana M Cuevas, Javier A Jacobo, Andres F Cardona-Zorrilla, Ivan A Bobadilla

**Affiliations:** 1 Radiotherapy Functional Unit, Centro de Tratamiento e Investigación sobre Cáncer (CTIC), Bogota, COL; 2 Research Group GIGA, Universidad del Bosque and Centro de Tratamiento e Investigación sobre Cáncer (CTIC), Bogota, COL; 3 Neuro-Oncology, Centro de Tratamiento e Investigación sobre Cáncer (CTIC), Bogota, COL

**Keywords:** adaptive motion management, advanced radiotherapy, sacral chordoma, sbrt (stereotactic body radiotherapy), stereotactic radiosurgery (cyberknife®)

## Abstract

Sacral chordoma is a rare and locally aggressive neoplasm that presents significant treatment challenges due to its anatomical location and tendency toward local invasion. Although complete surgical resection remains the gold standard for a potential cure, the high surgical morbidity necessitates alternative, less invasive treatment approaches. Robotic radiosurgery using the CyberKnife^®^ system has emerged as a promising option, particularly for patients who refuse surgery or are unsuitable surgical candidates. Furthermore, the precise delivery of high-dose radiation is crucial, as even minor sacral movements can affect dose distribution and treatment outcomes. We present the case of a 27-year-old male with a confirmed diagnosis of sacral chordoma who experienced progressive tumor growth. Given the high morbidity associated with surgical resection in the sacral region and the patient’s preference for avoiding surgery, a multidisciplinary team opted for stereotactic radiosurgery (SRS) using CyberKnife^®^. Robotic radiosurgery with real-time image guidance accounted for minor sacral movements during treatment delivery. The patient received 600-700 centigray (cGy) with a simultaneous integrated boost of radiation every other day, culminating in a total dose of 3,000 and 3,500 cGy to the planning target volume 30 and planning target volume 35, respectively, defined as the sacral mass with and without subclinical extension plus an appropriate margin. The quantitative analysis of intra-fractional patient movements recorded during treatment delivery revealed a range of displacements: 0.0-1.0 mm along the x-axis, 0.0- 1.5 millimeters (mm) along the y-axis, and 0.0- 1.0 mm along the z-axis. Rotational deviations were also assessed, with roll ranging from 0.0° to 0.8°, pitch from 0.0° to 0.4°, and jaw rotation from 0.0° to 0.7°. Following the completion of treatment on July 24, 2023, the patient reported a 90% reduction in pain. Subsequent clinical and radiological evaluations demonstrated substantial lesion regression with minimal toxicity, preserving the patient’s quality of life. This case reinforces the expanding body of evidence supporting advanced SRS for managing complex tumors. The successful application of CyberKnife^®^ in this sacral chordoma case, along with robust motion management strategies, highlights its potential as a safe and effective alternative when conventional surgery is not feasible.

## Introduction

Chordomas are rare malignant tumors originating from remnants of the notochord [1]. Although they typically exhibit slow growth, they are locally invasive and have a high recurrence rate, making them a therapeutic conundrum. Their management is further complicated by the proximity of their most common sites - the sacrum, skull base, and vertebral column - to critical neurovascular structures [1].

Sacral chordomas are among the most significant therapeutic challenges in musculoskeletal oncology [2]. While complete surgical resection is considered the only curative option, it is made difficult by the intricate anatomy and multiple biomechanical functions within the sacral region [2]. Thus, surgery in the sacral region is often associated with substantial morbidity, including neurological deficits, reduced quality of life (QoL), and functional impairment. Consequently, there is an increasing need for less invasive treatment modalities that can offer effective local control with reduced risk such as stereotactic radiosurgery (SRS) [3].

Radiosurgery is an advanced radiotherapy technique that enables highly precise treatment of tumors by allowing for the delivery of a high dose of radiation to a well-defined target. Systems such as CyberKnife® (Accuray, Inc., Sunnyvale, CA, USA) deliver high doses of radiation with sub-millimeter accuracy, even in anatomically complex regions [4]. These innovations align with reported trends, where the integration of image-guided treatment planning and adaptive dose delivery has significantly improved outcomes in challenging oncologic cases [4].

The need for precise treatment delivery to achieve effective local control is particularly important for sacral tumors. The imprecision of conventional external beam radiation, limitations in target immobilization techniques, and the need to administer high doses of radiation due to the radioresistant nature of chordomas have hindered the delivery of large radiation doses to this area [5].

Specifically, a critical consideration in treating sacral chordomas is motion control to prevent massive blood loss and potential neurological, bowel, and bladder function loss, which are common complications of sacral tumor resections [2]. Moreover, even minor sacral movements due to patient posture or involuntary muscular activity can compromise the accuracy of radiation delivery and change the dose distribution. While manufacturers of gantry-based treatment systems have integrated imaging elements, such as kilovolt (kV) imaging, megavolt (MV) imaging, and cone beam computed tomography (CT), to address patient setup errors common in traditional radiation therapy by enabling adjustment of the treatment couch during setup through two dimensions (2D) or three-dimensional (3D) image analysis, patient positions are not monitored during treatment, leading to movement uncertainty. Therefore, strong motion management strategies, such as real-time tracking and advanced immobilization techniques, are crucial. A non-invasive solution is also necessary, such as a fiducial-free tumor tracking algorithm.

Traditional tumor tracking often involves invasive fiducial implantation, which can be painful and may result in fiducial migration. CyberKnife® has provided a noninvasive solution by introducing a fiducial-free tracking algorithm for spine lesions.

Furthermore, while previous algorithms are effective for tumors near the vertebrae, they do not address pelvic areas due to the distance from the vertebral bodies, complicating the tracking of lower sacral lesions. However, it has been demonstrated that CyberKnife® can overcome this limitation and expand treatment options for patients with chordoma [2].

In this case study, we report the successful treatment of a young sacral chordoma patient using SRS based on a fiducial-free tracking algorithm: CyberKnife®. Drawing on previous research [2], we employed the center of the setup as a reference point for tracking the lower lumbar spine. We highlight the good clinical outcomes and the intricate technical details that ensured precise treatment delivery. Introducing this cutting-edge technology in a middle-income country like Colombia empowers patients with access to advanced and previously unattainable functional preservation options that significantly enhance their QoL.

## Case presentation

Patient demographics and clinical history

In April 2023, at the Centro de Tratamiento e Investigación sobre Cáncer (CTIC) in Bogotá, Colombia, a 27-year-old male, an environmental engineer, from Bogotá presented with periodic intense pain in the sacrum, measured as 8-9 on the visual analog scale (VAS), which caused significant discomfort and limited his daily activities. A previous histopathological examination, following a computed tomography-guided biopsy, confirmed the diagnosis of chordoma with infiltrative features. He had no significant comorbidities, and his overall performance was excellent (Eastern Cooperative Oncology Group Performance Status [ECOG] 1; Karnofsky Performance Status 90/100). He periodically experienced intense pain in the sacrum, measured as 8-9 on the VAS, which caused significant discomfort and limited his daily activities. The patient had undergone a biopsy in 2021, which resulted in a diagnosis of chordoid chordoma. The initial tumor size was 3 cm x 3 cm. Different external teams had evaluated his case and proposed radical surgery as the only option, but he declined it after careful consideration of the associated risks, including functional impairment.

Clinical examination and diagnostic test evaluation

**Figure 1:**
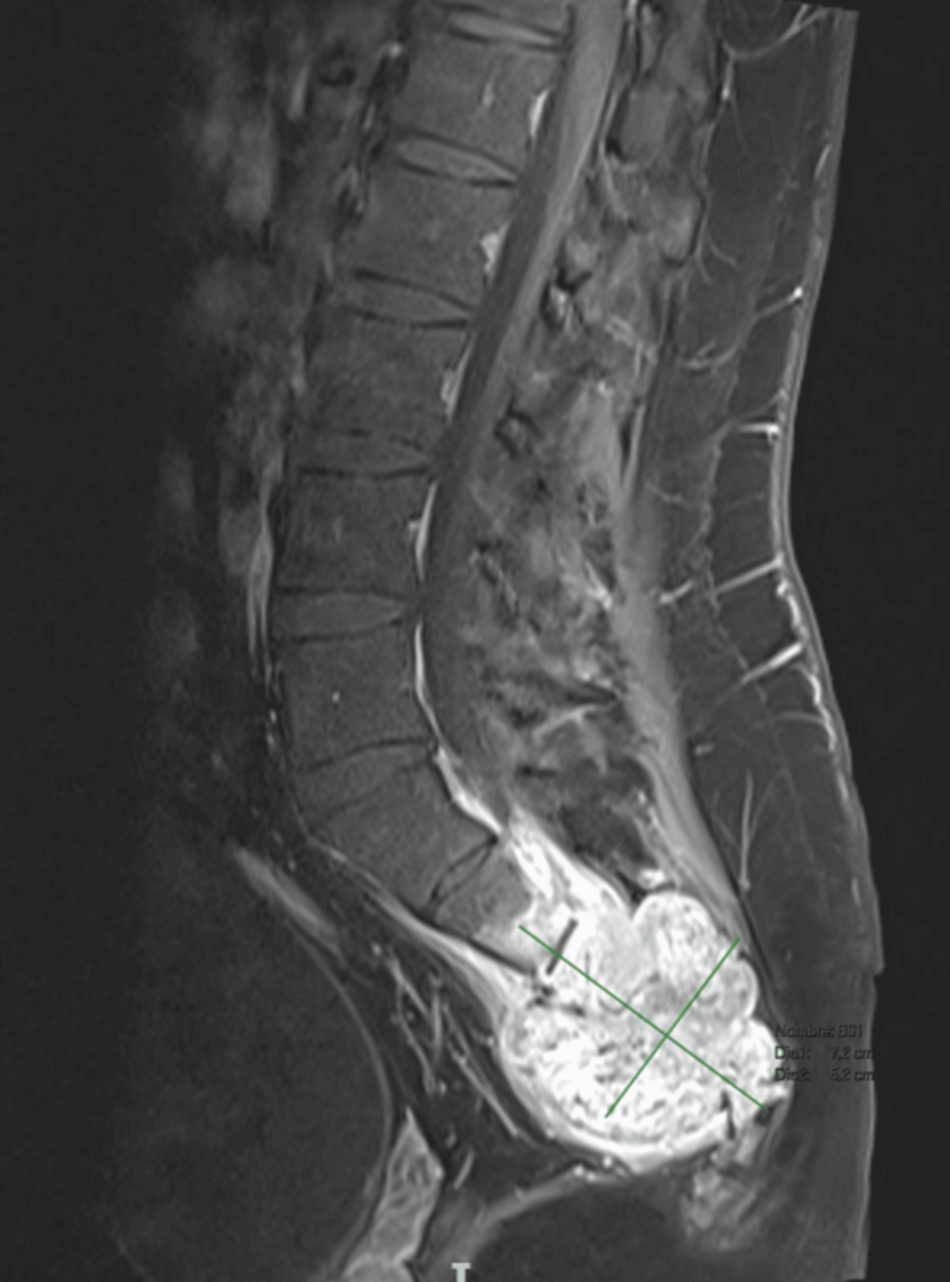
Contrast-enhanced T1-weighted MRI sequence. The sacral mass measured 7.2 cm × 5.2 cm.

Physical examination revealed localized pain in the sacral area without neurological deficits. Contrast-enhanced magnetic resonance imaging (MRI) of the sacrum, performed on June 29, 2023, revealed a diffusely infiltrative lesion involving the sacral segments S3-S4, extending into the coccygeal segments and adjacent soft tissues. The lesion measured approximately 7.2 cm × 5.2 cm (Figure 1).

The patient was evaluated by a multidisciplinary neuro-oncology team at CTIC, which confirmed progressive lesion growth in the sacrum since 2021. Treatment options were discussed with the patient, but the patient again declined surgery due to its risks, including functional impairment. Robotic radiosurgery was proposed as an alternative treatment.

Treatment planning and management

Simulation and Motion Management

During the simulation, the patient was placed in a supine position with his legs directed toward the gantry, with a cushion placed under his legs for added comfort, and a CT scan a 1-mm slice was performed in a SOMATOM go.Open Pro® (Siemens Healthineers, Erlangen, Germany). To prevent foods that cause rectal distention, which could impact the radiotherapy simulation and treatment, the patient was advised to follow a diet excluding milk, grains, and high-fat foods. This dietary regimen began one week before the simulation and continued throughout the radiosurgery treatment. Given the proximity of the sacral tumor to adjacent structures and nerves, high-precision motion management was essential. Thus, the treatment plan combined CT-based simulation and MRI, with robotic real-time image guidance using CyberKnife®. CyberKnife’s adaptive tracking algorithms would compensate for slight sacral movements caused by patient positioning and shifts in internal organs during each treatment session, ensuring accurate delivery of high-dose radiation to the defined planning target volume (PTV).

Radiotherapy Plan and Delivery

**Figure 2:**
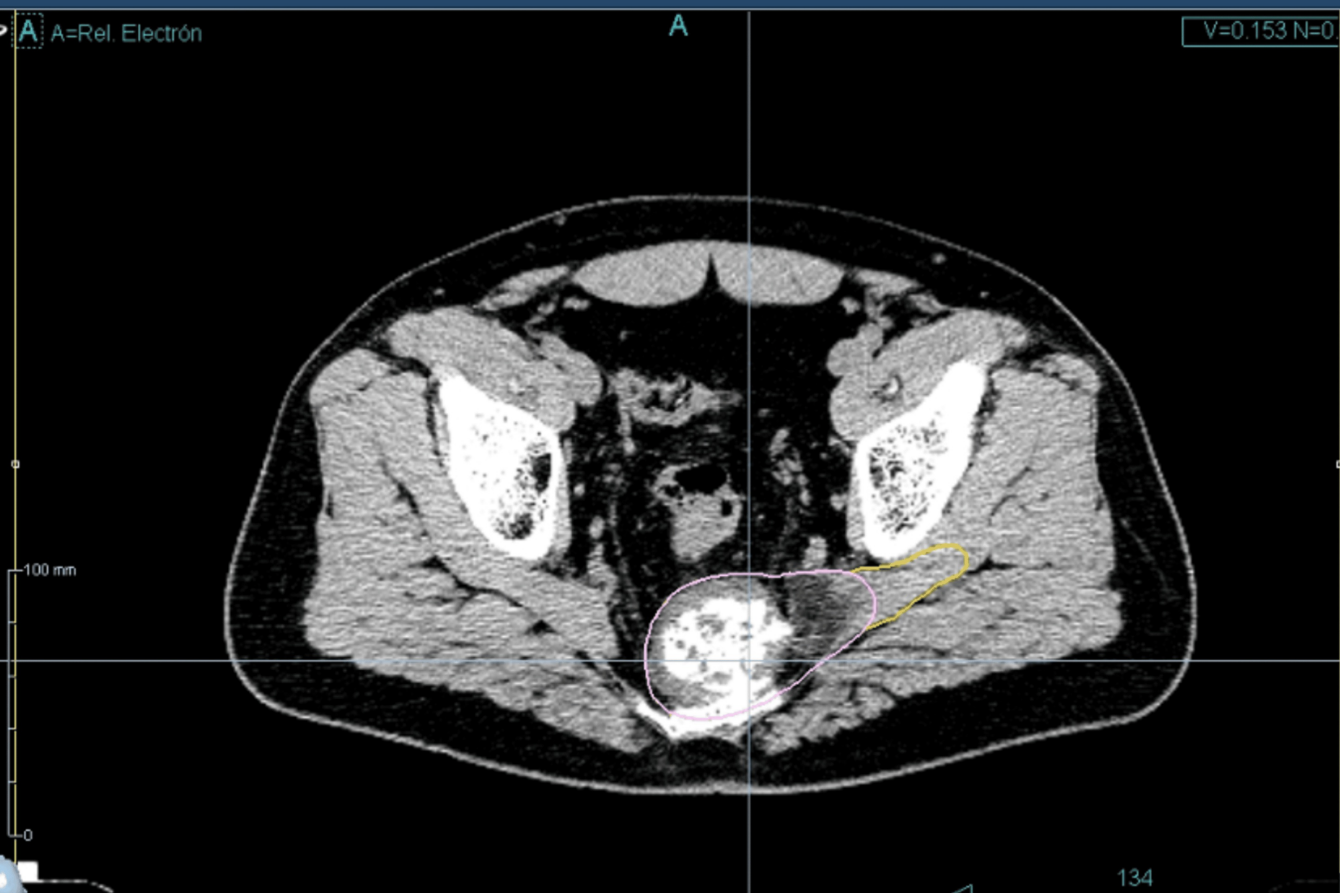
Radiotherapy volumes The gross tumor volume (pink) and the clinical target volume with the soft tissue extension (yellow).

**Figure 3:**
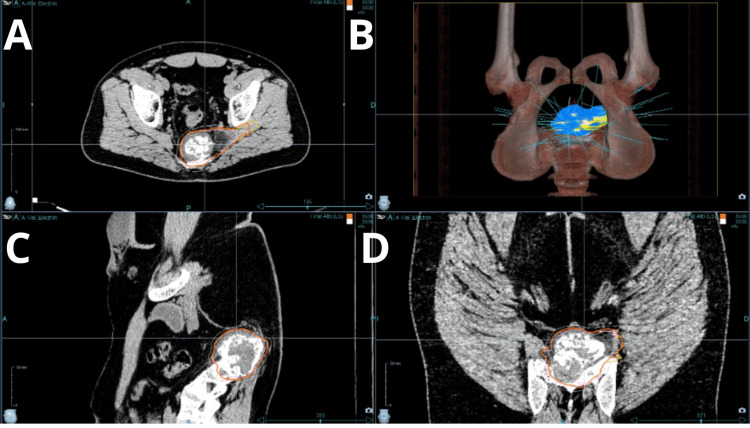
Robotic radiosurgery plan delivered by CyberKnife®. The dose is delivered and carefully tailored to the PTV and CyberKnife® plan with multiple beams. PTV, planning target volume

After thorough multidisciplinary evaluation and patient counseling, the decision was made to proceed with robotic real-time image guidance radiosurgery using CyberKnife® with a multileaf collimator (MLC) InCise 2® (Accuray, Inc.). For target delineation, CT and MRI images were fused. The contoured volumes included a gross tumor volume (GTV), representing the gross tumor identified on the CT and MRI, and a clinical target volume (CTV) consisting of the GTV plus areas of edema and soft tissue extension (Figure 2), including invasion through the central sciatic hole. Two PTVs were defined: the first PTV_30, receiving 3,000 centigray (cGy), was a 5-mm expansion of the CTV, and the second PTV_35, receiving 3,500 cGy, corresponded to the GTV without a margin. The plan involved SRS with an integrated simultaneous boost (Figure 3). The delivery was carefully monitored, and the daily imaging confirmed accurate dose placement. The radiotherapy plan was carried out using Precision Version 3.3.1.2® (Accuray, Inc.). There were 68 beams. The treatment was completed on July 24, 2023. The mean duration of five sessions was 21.9 minutes. Dose-volume histograms were calculated for the target lesions and all critical structures. The conformity index, homogeneity index, and coverage were used to evaluate the radiation dosimetry. A maximum punctual dose (0.03 cc) of 3.161 cGy for the cauda equina was achieved (tolerance set at 3,200 cGy) (Table 1).

**Table 1:** Planning objectives or constraints and achieved values PTV, planning target volume

Region of interest	Objective/constraint	Achieved value
PTV_30 (including the volume at risk for microscopic tumor invasion)	D95% ≥ 30 Gy	D97%: 30 Gy
PTV_35 (without the volume at risk for a microscopic tumor invasion)	D95% ≥ 35 Gy; Dmax ≤ 45.50 Gy	D95%: 35 Gy; Dmax: 41.72 Gy
Cauda equina	D0.03 cc < 32 Gy	D0.03 cc: 31.61 Gy
Bladder	D0.1 cc < 38 Gy	D0.1 cc: 9.66 Gy
Rectum	D0.1 cc < 38 Gy	D0.1 cc: 24.95 Gy
Femoral head	D10 cc < 30 Gy	Right femoral head D10 cc: 10.43 Gy; left femoral head D10 cc: 6.03 Gy
Bowel bag	D0.1 cc < 38 Gy	D0.1 cc: 37.81 Gy

Detailed Analysis of the Displacement Data

CyberKnife® is a robotic, image-guided SRS system equipped with a 6-MV linear accelerator mounted on a fully articulated robotic arm capable of translational and rotational movements to target tumors without needing rigid external fixation. Real-time image guidance was achieved by monitoring the patient’s position every 15-20 seconds. Translations and rotations were aligned during the patient setup and automatically corrected during the treatment delivery. Quantitative analysis of intrafractional patient movements recorded during the treatment delivery revealed mean translational displacements of 0.35 mm along the x-axis (range: 0.0-1.0 mm), 0.33 mm along the y-axis (range: 0.0-1.5 mm), and 0.33 mm along the z-axis (range: 0.0-1.0 mm). Rotational deviations were also assessed, showing a mean roll of 0.16° (range: 0.0°-0.8°), a pitch of 0.10° (range: 0.0°-0.4°), and a jaw rotation of 0.21° (range: 0.0°-0.7°).

Follow-up and outcome

**Figure 4:**
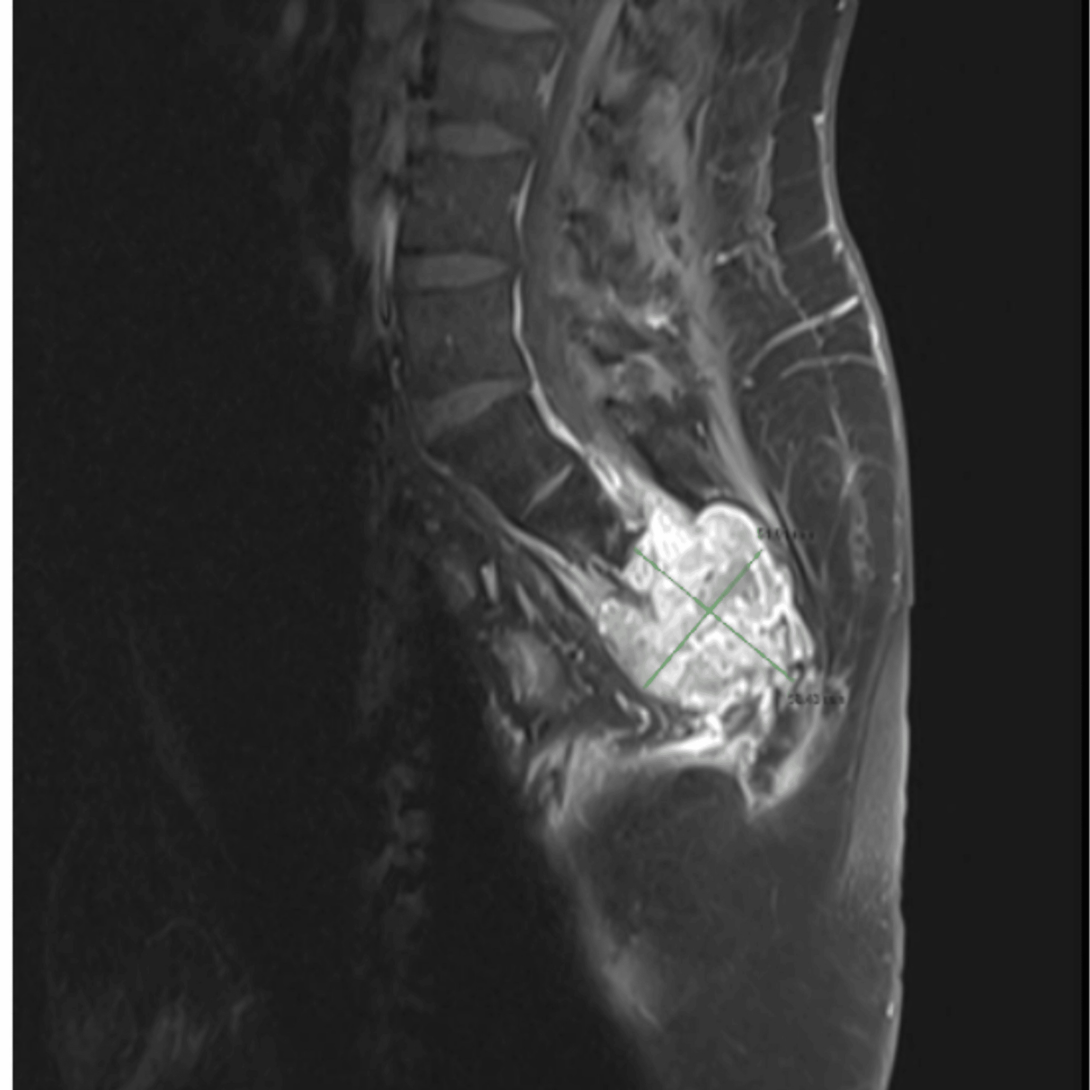
Enhanced-contrast MRI after the treatment. Sacral mass measuring approximately 5.8 cm × 5.1 cm.

**Figure 5:**
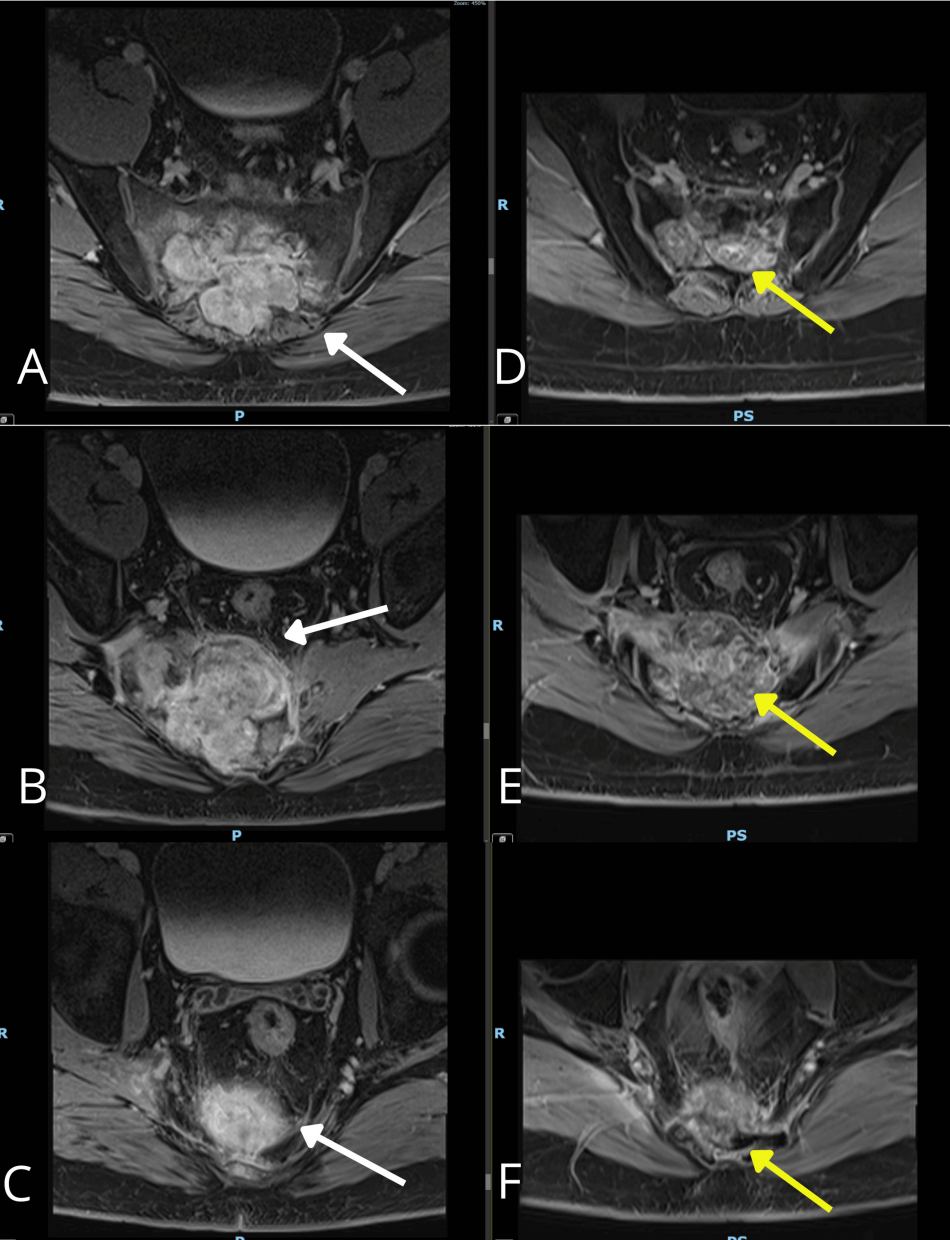
Enhanced-contrast MRI images of the sacral tumor. (A-C) Before the treatment in 2023. (D-F) After robotic radiosurgery in 2024. The white arrow in A, B, and C indicates the sacral mass before treatment. The yellow arrow highlights the reduction in the size of the sacral mass and the increasing separation from the rectum after treatment.

The follow-up MRI performed on June 20, 2024, demonstrated marked tumor regression, with the lesion measuring approximately 5.8 × 5.1 cm (Figure 4). Figure 5 illustrates the progressive reduction in the mass on MRI.

The patient experienced only mild, transient radiodermatitis in the sacral region, graded as grade 2 according to the Common Terminology Criteria for Adverse Events (CTCAE) version 5, which was effectively managed with topical treatments. Following the completion of radiotherapy on July 24, 2023, the patient maintained full neurological function and reported a significant reduction in pain (approximately 90% improvement). Since October 2023, his VAS rating has remained at 1 out of 10, with occasional episodes when it even reached 0.

**Figure 6:**
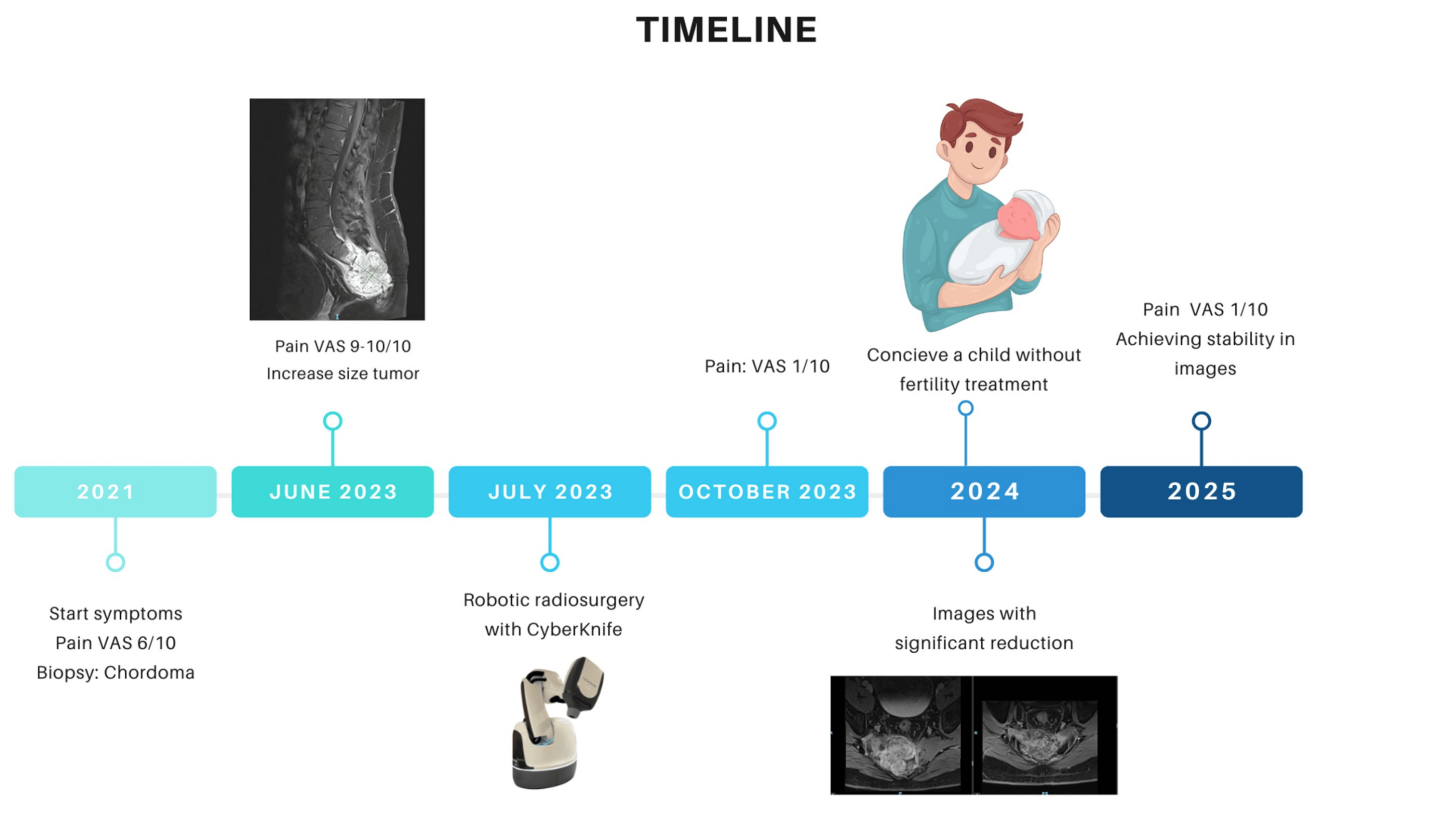
Timeline of events and follow-up. Robotic radiosurgery with CyberKnife image is an original work by Alejandro Gonzalez-Motta. Conceive a child without fertility treatment image provided by Canva. Creator: Heni Silvia. Used under Canva Pro license. Figure credit: Alejandro Gonzalez-Motta. VAS, visual analog scale

In 2024, the patient successfully preserved his sexual and reproductive function and conceived a child without the need for medical assistance. The latest MRI follow-ups in March 2025 revealed no changes in the lesion since the June 2024 imaging. As of 2025, the patient continues to have an excellent QoL (Figure 6).

## Discussion

The management of sacral chordoma remains challenging due to its infiltrative nature and the complex anatomy of the sacral region. While surgical resection is traditionally viewed as the only curative option, the high morbidity associated with sacral surgery often limits its feasibility.

Recent advances in SRS have significantly enhanced the management of chordomas, particularly for lesions in anatomically challenging locations where conventional surgery may lead to considerable morbidity. Studies have demonstrated that SRS can achieve local control rates of approximately 70-80% within two years of treatment while maintaining a favorable toxicity profile that helps preserve neurological function and overall QoL [6,7]. The precision of SRS - enabled by advanced image-guided planning and adaptive delivery techniques - allows for administering high ablative radiation doses directly to the tumor while minimizing the exposure of adjacent critical structures, such as the spinal cord and peripheral nerves. This targeted approach reduces the risk of radiation-induced complications, such as plexopathy, and plays a crucial role in preserving motor and sensory functions [8,9]. Collectively, these data underscore the potential of SRS as a noninvasive, function-preserving treatment modality for chordoma patients, particularly in cases in which extensive surgery is unfeasible or could result in significant functional impairment.

The alpha/beta (α/β) ratio is a measure of tumor sensitivity to varying dose-per-fraction (i.e., radiation dose per treatment session) radiotherapy regimens. Tumors with a low α/β ratio (<4 Gy) are more responsive to the effects of high-dose single sessions [10]. Based on historical studies and institutional experience, Georgetown University proposed an α/β ratio of 2.45 Gy for chordomas [11] and the same ratio for chondrosarcomas. This low α/β ratio for these tumors predicts improved outcomes of their treatment with high-dose hypofractionation or radiosurgery regimens [11].

In the case reported in this paper, extracranial fractionated radiosurgery allowed for the precise delivery of a high cumulative radiation dose while sparing adjacent critical structures. The integration of real-time image guidance and adaptive motion management was crucial, as even minimal sacral movements could compromise treatment accuracy and risk inducing sacral nerve lesions. The favorable clinical outcome observed - marked by significant symptomatic improvement and tumor regression - highlights the importance of providing patients with precise, high-technology radiation treatments for this type of tumor. Such treatments enable patients to lead fulfilling lives in both the sexual and neurological domains. Radiosurgery provided this patient a treatment option that not only preserved his functionality but also ensured an adequate QoL.

QoL is a crucial consideration in oncology and is now recognized as a critical outcome that directly influences treatment adherence and patient satisfaction [12]. Unlike survival alone, QoL reflects a multidimensional state encompassing physical functionality, emotional well-being, and social roles [13]. Both the cancer itself and its therapies can significantly diminish these aspects; for example, cancer survivors often experience a marked decline in health-related QoL as a result of the disease burden and the adverse effects of treatments [14]. The malignancy may cause debilitating symptoms (e.g., pain and fatigue) and psychological distress (e.g., anxiety and depression) that impair daily functioning and disrupt social participation. At the same time, interventions such as surgery, chemotherapy, and radiotherapy frequently introduce side effects that further compromise patients’ functional status. Notably, many of these treatment-related toxicities can persist long after the therapy is completed [15], underscoring the risk of chronic sequelae that can affect survivors’ well-being. Therefore, maintaining QoL is paramount, and there is a growing emphasis on treatment approaches that prolong survival, preserve functional status, and minimize long-term sequelae. In this context, robotic radiosurgery using the CyberKnife® system represents a paradigm shift in care for patients with complex tumors, such as sacral chordoma. By delivering highly conformal radiosurgery with real-time motion tracking, CyberKnife® can reduce morbidity while preserving neurological, sexual, and physical function, which are key elements of QoL. In the case reported in this paper, the patient demonstrated significant symptom relief while maintaining both his neurological and reproductive functions, which collectively contributed to a markedly improved post-treatment QoL. This outcome highlights the critical role of function-preserving, precision-based therapies in the management of oncological patients, especially in scenarios in which curative surgical interventions bear a considerable risk of resultant disability. In alternative circumstances, this patient might have experienced challenges in conceiving a child or functional impairments that limit physical movement, stemming from neurological and anatomical complications associated with surgical interventions. Such outcomes impose significant burdens that can profoundly alter the patient’s life experience, often in irreversible ways.

The analysis of intrafractional movements in this study revealed that even millimetric displacements could critically impact functional outcomes, especially in treatment scenarios with steep dose gradients. These findings underscore the inherent challenges of maintaining precise target coverage while sparing adjacent healthy tissues during SRS. They also highlight the need for a highly accurate motion management system capable of real-time monitoring and correction to mitigate potential dosimetric uncertainties.

The CyberKnife® system is a compelling option due to its integrated image guidance and continuous motion-tracking capabilities. Its ability to dynamically adjust for real-time intrafractional movements helps ensure that the radiation beam remains precisely aligned with the target throughout treatment delivery. This real-time adaptive correction minimizes the risk of misalignment caused by even minimal translational or rotational shifts, thereby enhancing treatment accuracy and safety. Therefore, implementing CyberKnife® technology in clinical practice could provide a critical advantage in managing the delicate balance between adequate tumor coverage and protecting critical structures, particularly in complex cases such as sacral chordoma.

This case also emphasizes the importance of a multidisciplinary approach in managing rare tumors. Close collaboration among neurosurgeons, radiation oncologists, medical physicists, and radiologists is essential in developing individualized treatment plans tailored to tumor biology and technical challenges. Further prospective studies and long-term follow-up are needed to refine these techniques and validate their efficacy in larger cohorts of sacral chordoma patients.

## Conclusions

In conclusion, the findings of this case report underscore the potential of extracranial fractionated radiosurgery using the CyberKnife® system as a viable treatment alternative for sacral chordoma patients who are either not candidates for or refuse surgical intervention. The integration of advanced motion management and real-time imaging dramatically enhances the precision and effectiveness of the treatment, leading to meaningful tumor regression with minimal side effects. Consequently, this technique signifies a critical advancement in SRS, highlighting its essential role in the comprehensive management of complex oncological conditions and offering hope and improved outcomes for challenging cases.
